# Glucagon-like Peptide-1 Acts as Signaling Mediator to Modulate Human Sperm Performance via Targeting Akt, JNK and IRS-1 Cell Signaling Cascades: Novel Insights into Sperm Physiopathology

**DOI:** 10.3390/jcm12113844

**Published:** 2023-06-04

**Authors:** Roberto Castiglione, Adele Vivacqua, Marta Santoro, Daniela De Rose, Giuseppina Peluso, Salvatore Panza, Saveria Aquila, Rosario D’Agata

**Affiliations:** 1 Department of Experimental and Clinical Medicine, University of Catania, 95123 Catania, Italy; giannitalia51@tiscali.it; 2 Department of Pharmacy and Science of Health and Nutrition, University of Calabria, Arcavacata di Rende, 87036 Cosenza, Italy; adele.vivacqua@unical.it (A.V.); ms83.santoro@libero.it (M.S.); daniela.derose@unical.it (D.D.R.); sasapanza@libero.it (S.P.); saveria.aquila@unical.it (S.A.); 3 Centro Sanitario, University of Calabria, Arcavacata di Rende, 87036 Cosenza, Italy; 4 Unit of Physiophatology of Reproduction, Annunziata Hospital, 87100 Cosenza, Italy; pina.peluso@libero.it

**Keywords:** GLP-1 effects, human ejaculated sperm, Akt, IRS-1, JNK

## Abstract

Recent evidence suggests that the male gonad is a potential target of glucagon-like peptide-1 (GLP-1). We investigated the effects of glucagon-like peptide-1 (GLP-1) on sperm function and the molecular mechanisms through which it may act. Semen samples of healthy men were incubated in the presence or absence of a GLP-1 mimetic analog, exendin-4 (Exe). In a different analysis, sperm were exposed to tumor necrosis factor (TNF-α) alone and, in some tubes, TNF-α was added after previous exposure to exendin-4 (Exe). Sperm parameters and protein-kinase B (p-Akt), insulin receptor substrate-1 (p-IRS-1 Ser312), and c Jun N-terminal protein kinase (p-JNK Thr183/Tyr185) were considered and evaluated. Sperm parameters, when incubated for 4 h in a simple defined balanced salt solution lacking protein, declined progressively with incubation time. The maximum decline was associated with a significant decrease in phosphorylated protein kinase B (p-Akt), concomitantly to an increase in insulin receptor substrate-1 (p-IRS-1 Ser312) and c Jun N-terminal protein kinase (p-JNK Thr183/Tyr185). Preincubation with exendin-4 (Exe) prevented this decline and maintained sperm motility (progressive—PM and total—TM). TNF-α exposure resulted in decreased sperm motility (PM and TM) and viability (V) in a concentration-dependent manner. Exe addition attenuated this TNF-α negative effect on sperm parameters. Glucagon-like peptide-1 (GLP-1) also acts by reducing levels of the “negative” kinases p-IRS-1Ser312 and p-JNK. An imbalance involving these three kinases in sperm, as it occurs in somatic cells, is a novel scenario that may participate in sperm physiopathology.

## 1. Introduction

Glucagon-like peptide-1 (GLP-1) is an incretin hormone released by enteroendocrine L cells in the intestine in response to food ingestion, and GLP-1 effects first include higher insulin release in response to glucose [1]. However, the presence of this receptor is not limited to the pancreatic cells, as it is expressed in the heart, lungs, kidneys, and adipocytes [2,3,4]. Recent evidence suggests that the male gonad is a potential target of glucagon-like peptide (GLP-1) action. Male glucagon-like peptide-1 (GLP-1) receptor knockout mice exhibit a reduced seminal vesicle and testis weights compared to control animals [5]. Recently, we detected the receptor for GLP-1 in human sperm and demonstrated that activation of this receptor is important in the regulation of sperm energy homeostasis by acting on different metabolic branches [6].

It is known that insulin is the key hormone for glucose homeostasis in our body and in human sperm. Our previous results indicated that GLP-1 has a potential effect on the insulin signal in these cells [6].

In vitro findings strengthen the important role of insulin in sperm physiology; insulin is expressed and secreted by human sperm, provides autocrine regulation of sperm metabolism [7,8], and it is able to reduce mitochondrial reactive oxidative species (ROS) production and total caspase activity [7]. Insulin receptor substrate-1 (IRS-1) is the principal substrate that is phosphorylated on multiple tyrosine residues after receptor activation, creating an active signaling complex [9], then it binds to the PI3K/Akt protein complex, a major pro-survival pathway in the cells. Akt promotes various biological properties, including motility, morphology, and survival changes in human sperm (“positive kinase”) [6,7,10]. On the other hand, modification to serine/threonine phosphorylation of IRS-1 leads to inhibition of this pro-survival complex. Indeed, in humans, heterologous mechanisms that converge to activate IRS-1 phosphorylation at inhibitory Ser 312 site (“Ser 307 in animals”), which is a poor insulin receptor substrate in transducing insulin signal, bringing about a status of insulin resistance [11,12]. Tumor necrosis factor (TNF-α) is also a pro-apoptotic inducer in human sperm (“negative kinase”) [13]. The biological activities of this cytokine are exerted through the activation of multiple downstream signaling effectors, including c-Jun N-terminal protein kinase (p-JNK).

Importantly, glucagon-like peptide-1 (GLP-1) exerts antiapoptotic effects [14,15], counteracting cytokine action and gene expression [16,17]. The activation of GLP-1R impairs proapoptotic signaling at the level of c-Jun N-terminal protein kinase (JNK) and reduces the inhibition of the IRS-1/Akt pathway by preventing inhibitory serine residue phosphorylation of IRS-1 proteins in insulin-secreting cells [16].

The recent detection of GLP-1 receptor (GLP-1R) in human sperm, taken together with the demonstration that agonist addition improves the function and affects the metabolism of human sperm [6], implicates a role for glucagon-like peptide-1 (GLP-1) in human sperm signaling pathways, at least in vitro. This prompted us to deepen the mechanisms by which this peptide may act on sperm.

## 2. Materials and Methods

### 2.1. Reagents

Earle’s balanced salt solution (EBSS) medium was obtained from Gibco (Life Technology, Milan, Italy). Dimethyl sulfoxide (DMSO), wortmannin 12–338, which blocks the catalytic activity of PI3-kinase without affecting the upstream signaling events, exedin-4 (Exe), a specific GLP-1R agonist, and all other chemicals were purchased from Sigma-Aldrich (Milan, Italy). Acrylamide bisacrylamide was obtained from Labtek Eurobio (Milan, Italy). Triton X-100 was purchased from Farmitalia Carlo Erba (Milan, Italy). The ECL Plus Western blotting detection system, Hybond ECL TM, and HEPES sodium salt were purchased from Amersham Pharmacia Biotech (Amersham, Buckinghamshire, UK). Anti-p-AKT (p-Ser473), total anti-AKT and anti-β-actin antibodies, Exe, peroxidase-coupled anti-rabbit immunoglobulin G (IgG), peroxidase-coupled anti-mouse IgG, anti-mouse and anti-rabbit fluorescein isothiocyanate (FITC)-conjugated antibodies, and normal mouse and normal rabbit sera were obtained from Santa Cruz Biotechnology (Heidelberg, Germany). Anti-SAPK/JNK and anti-phospho-SAPK/JNK (Thr183/Tyr185) were purchased from Cell Signaling Technology (Euroclone, Milan, Italy); anti-IRS-1 (MW171), and anti-phospho IRS1 (Ser312) antibodies were purchased from Abcam (DBA, Milan, Italy).

### 2.2. Semen Samples and Sperm Preparations

Human semen samples were obtained from 45 healthy normospermic men according to the WHO Sixth Edition [18], undergoing semen analysis as part of their fertility evaluation. Semen samples in the study groups were collected in sterile plastic jars through masturbation after 3–5 days of abstinence. Semen with >1 × 10^6^/mL leukocytes at microscopic evaluation were discarded. After liquefaction at 37 °C, three different semen samples, were pooled to compensate for individual variation and purified by swim-up, performed as previously described [6]. The selected human sperm were then diluted in EBSS supplemented on the day of use with 3 mg/100 mL sodium pyruvate, 10 mM/100 mL NaHCO_3_ and 0.37 mL/100 mL of 60% (*v*/*v*), sodium lactate syrup, and antibiotics. For all experiments in this study, sperm suspensions (0.5 mL/tube) were adjusted to a concentration of 10 × 10^6^ cells/tube and remained untreated as a control (C) or exposed to a specific amount of the relative drug according to the experimental design. All vehicles of drugs used in the experiments were initially tested for cytotoxic effects on sperm and deleterious effects were not observed. DMSO at 0.012% final concentration (used to dilute Exe and wortmannin), when used as solvent controls, did not induce any positive results in all in vitro assays performed. For the sake of simplicity, the 300 pM Exe and 0.1 ng TNF-α are here referred to as low and 50 nM Exe and 1 ng TNF-α as high concentrations. In all experiments, sperm were incubated at 37 °C under CO_2_ for the specified time. For the time course, the cells were incubated for 1, 2, and 4 h; for the other experiments, they were incubated 1, 1 ½, 2 h, and 4 h.

### 2.3. Evaluation of Sperm Motility

Sperm motility was assessed by means of light microscopy examining aliquots of each sample in the absence (C) or in the presence of the indicated substances (treatments). Three independent observers scored at least 200 cells. Sperm motility was expressed as a percentage, which includes total sperm motility (TM) (percentage of sperm that exhibit motility of any form) and progressive sperm motility (PM) (percentage of sperm that exhibit rapid, linear movement).

### 2.4. Evaluation of Sperm Viability

Sperm viability was assessed by the red eosin exclusion test using Eosin Y [18]. An independent observer scored 200 cells for stain uptake (dead cells) or exclusion (live cells).

### 2.5. Western Blot Analysis of Sperm Protein

Swim-up-selected sperm samples at the end of incubation were washed twice with EBSS, incubated for 30 min (min) at 37 °C under 5% CO_2_, and then centrifuged for 5 min at 1000× *g*. The pellet was resuspended in lysis buffer (62.5 mmol/l Tris-HCl, pH 6.8; 150 mM NaCl; 2% sodium dodecyl sulfate; 1% Triton X100; 10% glycerol; 1 mM phenylmethylsulfonyl fluoride; 10 μg/mL leupeptin; 10 μg/mL aprotinin; 2 μg/mL pepstatin), as previously described [19]. Protein bands were quantified by scanning densitometry (Imaging Densitometer GS-700 Bio-Rad, Milan, Italy), evaluated in terms of arbitrary densitometric units and presented as means ± SEM. As internal controls, all blotting membranes were subsequently stripped (glycine 0.2 M, pH 2.6, for 30 min at room temperature) and re-probed with anti-β-actin antibody (1:1000) or total anti-Akt as a control for equal loading.

### 2.6. Data Analysis

The Western blot analyses were repeated three times on independent samples; columns show the summarized data of the band intensities (Arbitrary Units) after being normalized to β-actin or total AKT to ensure equal loading across all lines. Results from three or more experiments were expressed as the means ± SEM. Data were subjected to statistical analysis using one-way analysis of variance (ANOVA) and post hoc comparison of group means was performed by Fisher’s protected least significant difference (PLSD) test using Graph Pad Prism v. 6 (Graph Pad Software, La Jolla, CA, USA), and statistical significance was accepted when *p* < 0.05.

## 3. Results

### 3.1. Spontaneous Variation of Sperm Parameters and Kinase Activity over Time

**Figure 1:**
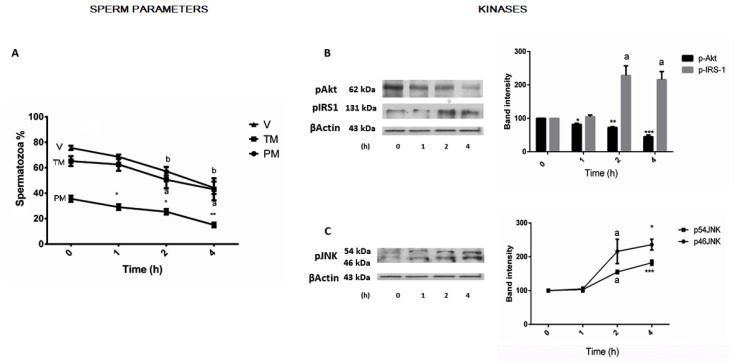
Effect of 4 h incubation time on sperm motility, viability, activated protein kinase B (p-Akt), activated insulin-substrate-1 (p-IRS1 ser312), and activated c-Jun N-terminal protein kinase (p-JNK isoforms) levels. (**A**)—Total (TM), progressive motility (PM), and vitality (V) are plotted as function of time. Data represent results of analyses replicated five times on independent samples. (**B**)—At the end of each indicated time sperm lysates were subjected to immunoblotting with specific antibodies, as described under Material and Methods. A representative immune-blot for p-Akt and p-IRS-1ser312 is shown (left) and similar results were obtained in two other independent experiments. The bar plots for p-Akt and p-IRS-1 ser312 show the summarized data of the band intensities (Arbitrary Units) after being normalized to β-actin (right). (**C**)—Representative immunoblot (left) for the p46 and p54 JNK isoforms. Results of band intensities of the three experiments are also shown (right). Each column shows mean ± SEM. Data were analyzed by one-way ANOVA (*p* < 0.02 for all panels) and post hoc comparison of means was determined using Fisher’s PLSD test. (**A**) * *p* < 0.0003 vs. 0; ** *p* < 0.0001 vs. 0; a *p* < 0.01 vs. 0; b *p* < 0.003 vs. 0. (**B**,**C**): * *p* < 0.008 vs. 0; ** *p* < 0.002 vs. 0; *** *p* < 0.0003 vs. 0; a *p* < 0.01 vs. 0.

The first step of this study was designed to gain insights into induced time-dependent variations in sperm parameters and the concomitant associated changes in kinases, which are recognized components of different cellular downstream signaling cascades. Focusing our interest on activated protein-kinase B (p-Akt), activated insulin receptor-substrate 1 (p-IRS-1ser312), and c-Jun N-terminal protein kinase (p-JNK isoforms), we incubated selected untreated sperm for 1, 2, and 4 h. We observed a progressive linear and significant parallel time-dependent decline in motility (TM-PM) and viability (V) (Figure 1A). Progressive motility started to decrease at 1 h: the largest drop, approximately 57% of the preincubation average value (PRE), was observed after 4 h. Decreases in total motility (≈34%) and cell viability (≈40%) were lower and similarly peaked at 4 h (see Figure 1 for details). This decline in sperm parameters was concomitant with a progressive time-dependent decline in p-AKT starting at 1 h (Figure 1B), with an almost undetectable band signal at 4 h and a significant time-induced rise starting at 2 h in the activated IRS-1 ser312 (≈1.2-fold) (Figure 1B, right) and activated JNK isoforms and, at 4 h, ≈1.4- and ≈0.8-fold for p-46 and p-54, respectively (Figure 1C, right).

### 3.2. Glucagon-like Peptide-1 (GLP-1) Is a Pro-Survival Factor That Preserves Sperm Motility and Viability and Acts Mainly but Not Only via the Protein Kinase B (Akt) Signaling Pathway

**Figure 2:**
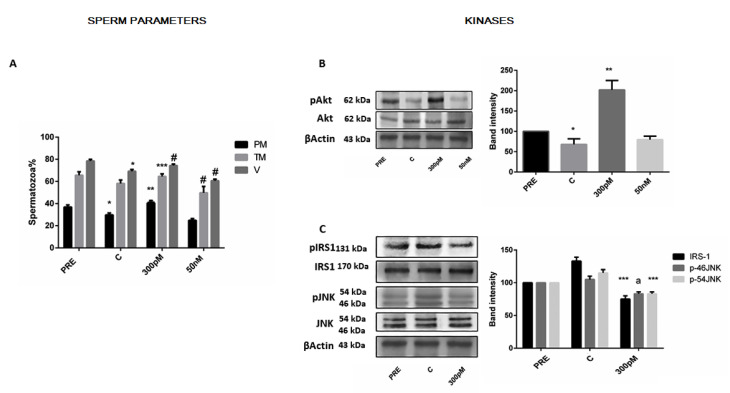
Impact of 300 pM Exe exposure for 1 h on sperm parameters, p-Akt, p-IRS-1 ser 312, and p-JNK isoform levels. (**A**)—Motility and vitality are plotted after Exe exposure. All data summarize results of experiments repeated 5 times on independent samples. (**B**)—WB analyses (left) of the effect of Exe on Akt phosphorylation status. The bar blots (right) show the summarized data of band intensities of experiments repeated on three independent samples. (**C**)—Western blot of p-IRS-1 ser 312 and p-JNK (left) after analog and results of quantification of multiple experiments by densitometric scan (right) are represented. Representative blot is presented and similar results were observed in two independent experiments. All data presented are mean ± SEM. The results were analyzed by one-way ANOVA (*p* < 0.05) and post hoc comparison of means was determined using Fisher’s PLSD test. (**A**): * *p* < 0.005 vs. PRE; ** *p* < 0.0006 vs. C; *** *p* < 0.004 vs. C; # *p* < 0.01 vs. C. (**B**,**C**): * *p* < 0.05 vs. PRE; ** *p* < 0.002 vs. C; *** *p* < 0.01 vs. C; a *p*< 0.05 vs. C. PRE, parameters at time zero incubation; C, untreated samples at the end of incubation.

**Figure 3:**
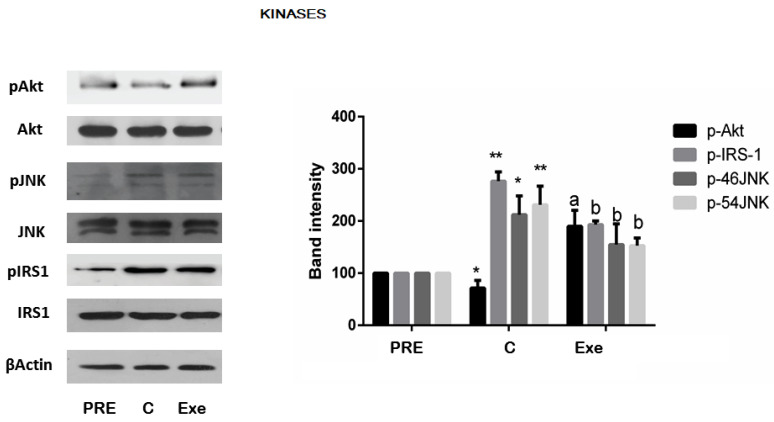
Effect of 300 pM Exe incubated for 2 h on activated kinase B (p-Akt), activated insulin substrate 1 (p-IRS-1 ser312), and activated c-Jun N-terminal protein kinase (p-JNK isoforms) levels. Representative blot analysis of the effect of Exe incubated for 2 h on p-Akt, p-IRS-1, and p-JNK isoforms (left). This experiment was repeated three times on independent specimens with similar results. The bar blot shows the summarized data of band intensities (right) and is expressed as mean ± SEM. Data were analyzed by one-way ANOVA (*p* < 0.01) and post hoc comparison of means was determined using Fisher’s PLSD test. * *p* < 0.05 vs. PRE; ** *p* < 0.007 vs. PRE; a *p* < 0.004 vs. C; b *p* < 0.02 vs. C. PRE, parameters at time zero incubation; C, untreated samples at the end of incubation.

Human sperm were incubated for 1 and 2 h with low or high concentrations of the natural and stable GLP-1 mimetic, exendin-4 (Exe). When sperm populations were incubated for 1 h, a low concentration rescued the decline in motility and viability and ameliorated sperm function such that motility and viability were unchanged from their pre-incubation levels (see details Figure 2A), although the degree of forward motility improvement in the whole study varied from sample to sample, ranging from 22 to 52% of control. This exedin-4 (Exe) concentration (300 pM) was chosen because it was the most effective to affect sperm motility in a previous concentration–response pilot analysis [6]. In contrast, the higher concentration of the agonist, surprisingly, did not stimulate at all either motility or viability (Figure 2A). The results of the analog effects on sperm parameters of the 2 h design, replicated and fit those of 1 h incubation. We then investigated molecular mechanisms mediating such effects. In the 1 h analysis, p-Akt levels in untreated cells were decreased, as previously detected in the time-course experiments. Concomitantly, the low exedin-4 (Exe) concentration resulted in an increase in protein kinase B phosphorylation (p-Akt) of ≈220% (Figure 2B). Since we concluded that Exe-induced improvement to sperm motility was mediated by activation of Akt, we speculated whether the negative impact on sperm by high-concentration Exe was due to inhibition of Akt phosphorylation. Confirming our hypothesis, the results revealed that exposure to this high concentration completely prevented the activation of protein kinase B phosphorylation (p-Akt) (Figure 2B). As previously observed in the time-course design, after 2 h of incubation (Figure 3), we observed an increase in the two negative kinases. Interestingly, the 300 pM concentration of analog led to a significant decrease in the basal phosphorylation of the two negative kinases tested, on average 30.7% and 31.2% for p-IRS-1 ser 312 and p-JNK isoforms, respectively (Figure 2C).

### 3.3. Inhibition of PI3K/Akt Complex Attenuated the Positive Effect of Exe on Sperm

**Figure 4:**
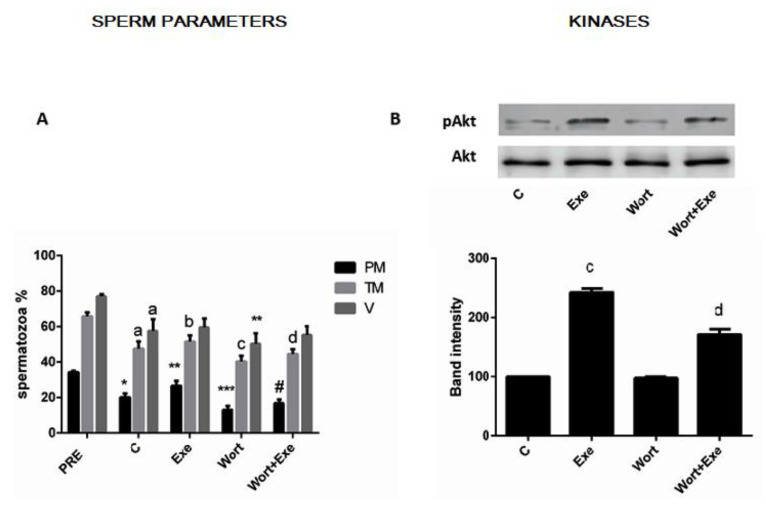
Effect of PI3K kinase inhibitor, wortmannin (Wort), upon proportion of motile and viable sperm and on Exe action and protein kinase B activation status (p-Akt). (**A**)—Motility and vitality after the inhibitor wortmannin (20µM), Exe (300 pM), and inhibitor (Wort) + agonist addition are plotted. (**B**)—Representative immunoblot of phosphorylated Akt after wort and Exe is shown and similar results were obtained in other experiments. The bar blot shows the summarized data of the band intensities as mean ± SEM, of experiments repeated five times (**A**) and three times (**B**) on independent samples. The band intensities of semen samples were here normalized to protein-kinase B (Akt). Comparisons among group means were conducted using Fisher’s PLSD test; (**A**,**B**): * *p* < 0.009 vs. PRE; ** *p* < 0.004 vs. C; *** *p* < 0.007 vs. C; a *p* < 0.002 vs. PRE; b *p* < 0.01 vs. C; c *p* < 0.02 vs. C; d *p* < 0.01 vs. Exe; # *p* < 0.002 vs. Exe. PRE, values at time zero incubation, C values of untreated samples at the end of 4 h incubation.

If Exe exerts its effect on sperm by activating the phosphatidylinositol kinase PI3K/Akt pathway, it follows that inhibition of this complex should abrogate or reduce analog action. To investigate if this was the mechanism, human sperm were incubated with wortmannin [10], a fungal metabolite which was shown to potently inhibit PI3-kinase, for 4 h, and sperm parameters and Akt phosphorylation activity were quantified. Inhibition of this complex resulted in a significant reduction in basal motility and viability, and significantly attenuated the positive effect of exedin-4 (Exe) on sperm parameters (Figure 4A). Concomitantly, an equally significant decrease in Exe-induced p-Akt level expression by ≈32% was detected (Figure 4B).

### 3.4. Exedin-4 (Exe) Pretreatment Partially Counteracts Negative Tumor Necrosis Factor (TNF-α)-Induced Effects in Sperm and, Reciprocally, TNF-α Attenuates Pro-Survival Effect of Exe

**Figure 5:**
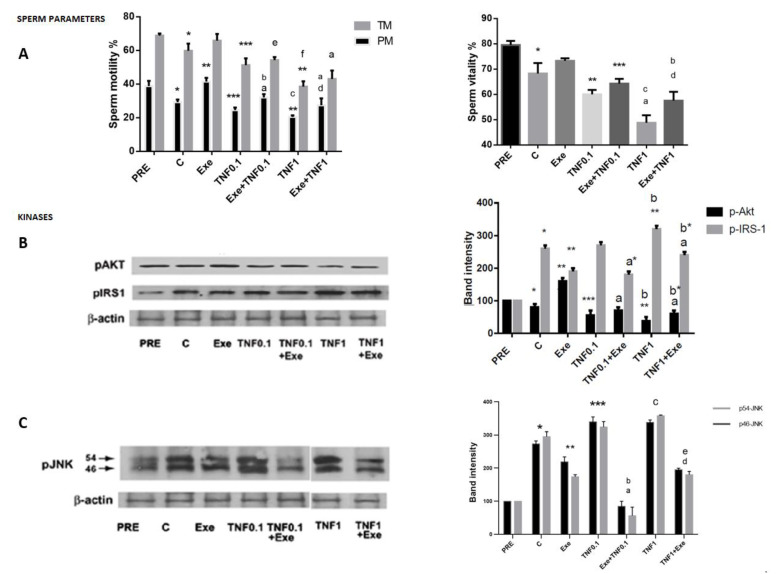
Interaction of Exe and TNF on parameters and signaling pathways of human sperm including activated kinase B (p-Akt), activated insulin substrate-1 (p-IRS-1 ser312), and activated c-Jun N-terminal protein kinase (p-JNK isoforms). (**A**)—Motility (PM, TM) (left) and vitality (V) (right) of human sperm incubated with Exe for 90 min in presence or absence of different doses of TNF-α as indicated, added after 45 min. (**B**)—Representative immunoblot of Akt and IRS-1 is shown (left) and similar results were observed in other independent samples with the quantitation of the band intensities (right). (**C**)—Left panel representative immunoblot of p-JNK isoforms and right panel summarized band intensities of analysis of independent measurements. Results are mean ± SEM of experiments repeated from three (**B**,**C**) to five measurements (**A**) performed with sperm samples from different subjects. Data were analyzed by one-way ANOVA. Comparisons among group means were conducted using Fisher’s PLSD test. (**A**)**—Left**: * *p* < 0.0001 vs. PRE; ** *p* < 0.0001 vs. C; *** *p* < 0.01 vs. C; a *p* < 0.0001 vs. Exe; b *p* < 0.0002 vs. TNF0.1; c *p* < 0.02 vs. TNF0.1; d *p* < 0.005 vs. TNF1; e *p* < 0.002 vs. Exe; f *p* < 0.0009 vs. TNF0.1. (**A**)**—Right**-: * *p* < 0.001 vs. PRE; ** *p* < 0.01 vs. C; *** *p* < 0.0001 vs. Exe; a *p* < 0.006 vs. C; b *p* < 0.0001 vs. Exe; c *p* < 0.0001 vs. TNF0.1; d *p* < 0.008 vs. TNF1. (**B**): * *p* < 0.001 vs PRE; ** *p* < 0.0001 vs. C; *** *p* < 0.009 vs. C.; a *p* < 0.0001 vs. Exe; a* *p* < 0.0001 vs. TNF 0.1; b *p* < 0.01 vs. TNF 0.1; b* *p* < 0.001 vs. TNF 1. (**C**)**—Right**: (statistic for both bars) * *p* < 0.01 vs. PRE; ** *p* < 0.001 vs. C; *** *p* < 0.002 vs. C; a *p* < 0.0001 vs. Exe; b *p* < 0.0001 vs. TNF 0.1; c *p* < 0.0002 vs. C; d *p* < 0.02 vs. Exe, only for p46 isoform; e *p* < 0.001 vs. TNF1. PRE, values at time zero incubation, C, values of untreated samples at the end of incubation.

Recent studies have demonstrated that glucagon-like peptide-1 (GLP-1) analog exposure abrogates the negative effect of tumor necrosis factor (TNF-α) on pancreatic islets [16,20]. Thus, we hypothesized whether this modulation of TNF-α’s effects by exedin-4 (Exe) affected also sperm. For this analysis, cells were incubated with a low Exe concentration for 90 min in the presence or absence of different concentrations of TNF-α added after 45 min in the appropriate tubes. As expected, TNF-α exposure led to a decline in progressive motility in a dose-dependent (0.1 and 1 ng) manner by 17% and 31%, respectively, compared to unexposed samples (Figure 5A left). The same effect was observed with total motility being lowered by 16.6% and 36.1% after low and high TNF-α concentrations (Figure 5 left), respectively, and on viability by 12% and 29%, respectively (Figure 5A right). These concentrations of TNF-α were chosen based on a previous concentration–effect pilot study [13] and results were consistent with our previous findings [13]. This TNF-α-dependent decline in sperm motility and viability could be partially rescued by Exe pre-exposure. Indeed, the results of this analysis indicated that the presence of Exe resulted in a significant reduction in the TNF-α-induced effects on progressive motility of ≈30.6% and ≈37.1% and, to a lesser extent, on total motility by ≈6% and ≈11% (Figure 5A left) and on viability by ≈7% and ≈18% (Figure 5A right) at low and high TNF-α concentrations, respectively.

### 3.5. Mechanism through Which Exendin-4 (Exe) May Exert a Protective Effect on Tumor Necrosis Factor (TNF-α) Action and Signaling Pathway in Sperm

To define the signaling mechanisms mediating tumor necrosis Factor (TNF-α) induced effects on sperm parameters, cell lysates were immunoblotted for the indicated phosphorylated kinases at the end of incubation. At the end of 90 min incubation of untreated samples, as previously observed in this study, levels of activated protein kinase B (p-Akt) were decreased and activated c-Jun N-terminal protein kinase (p-JNK) and insulin receptor substrate-1 (p-IRS-1 ser312) were both increased (Figure 5B,C). As expected, addition of Exe was associated with a significant increase in protein-kinase B phosphorylation (p-Akt) and with a concomitant and statistically significant reduction in phosphorylation time-induced levels of the two negative kinases (Figure 5B,C). Low and high TNF-α concentrations stimulated significant phosphorylation of the p-46 and p-54 JNK isoforms above control values (Figure 5C). Higher TNF-α concentrations (5 ng/mL) did not further activate JNK phosphorylation (personal observations). The effect of low TNFα concentration was fully abrogated and partially reversed by the high TNF-α concentration of ≈50% when cells were preincubated with a standard dose (300 pM) of Exe (Figure 5C). In contrast, TNF-α decreased basal p-Akt and nullified the stimulatory effect of Exe upon Akt phosphorylation (Figure 5B). Because TNF-α has been shown to phosphorylate serine residues on IRS-1 ser312 protein in multiple cells and tissues (11,12), we speculated whether this was also the case for male gametes. Indeed, exposure of sperm to high TNF-α concentration resulted in statistically significant increased phosphorylation of IRS-1 ser312 with respect to the control levels (Figure 5B). In the presence of exendin-4 (Exe), this effect of TNF-α was attenuated.

## 4. Discussion

Recently, we demonstrated the presence of glucagon-like peptide-1 receptor (GLP-1R) in human sperm and its activation by exedin-4 (Exe), which is involved in the PKA pathway [6]. Herein, we focused on the role of GLP-1R activation in sperm and presented novel insights into human sperm biology. It would appear that, when we used a simple defined balanced salt solution lacking protein to incubate populations of human sperm in vitro, they gradually lost motility and viability in a time-dependent fashion and the maximum decline was detected at 4 h. This impairment of sperm parameters was concomitant with a progressive decline in activated protein kinase B levels (p-AKT), a pro-survival enzyme, consistent with a previous study [10], in association with an increase in insulin receptor substrate-1 phosphorylation at Ser312 (p-IRS-1) and of activated c-Jun N-terminal protein kinase (p-JNK) isoforms considered to be proapoptotic inducers [11,12]. This novel finding lends support to the hypothesis that low-performing sperm during this prolonged period might also be due to an imbalance in the activity of opposing kinase downstream signaling pathways. Consequently, we reasoned that the equilibrium status among these signaling pathways may define the quality and healthy status of the sperm. The addition of 300 pM analog for 1 and 2 h rescued this decline in motility and viability and maintained swimming sperm at preincubation levels. This effect was associated with an important and significant activation involving the phosphorylation state of Akt at position Ser473 and, concomitantly, exedin-4 (Exe) triggered a reduction in the time-induced increase in insulin receptor substrate-1 phosphorylation at Ser312 (p-IRS-1) and activated c-Jun N-terminal protein kinase (p-JNK) activities. Thus, Exe addition restored the imbalance between p-Akt, p-IRS-1, and p-JNK isoform production. Thus, via this mechanism, GLP-1 is a pro-survival inducer in sperm. Surprisingly, a high dose of analog, 50 nM, elicited a decline in sperm motility and viability and notably negated Exe-dependent Akt activation. The reason for this is not clear; however, we cannot exclude that desensitization to such a large amount or a cytotoxic mechanism being behind this effect. Evidence has shown that the PI3K/Akt complex protects cells from entering an apoptotic-like state [21,22,23,24], and activation of this complex is associated with pro-survival effects on human sperm [6,7,10]. Additionally, DNA fragmentation was negatively correlated with the levels of phosphorylated Akt in patients with varicocele; the levels of activated Akt were also negatively correlated with the percentage of sperm head abnormalities (“positive kinase”) [25]. In contrast, the addition of inhibitors of this complex resulted in an apoptotic cascade characterized by motility loss, ROS generation, caspase activation, and oxidative DNA damage [22]. In this study, by using wortmannin, we obtained the same important and significant drop in baseline progressive motility and viability and fully negated the amelioration of sperm performance after Exe treatment via a significant attenuation to the positive inductive effect of Exe on Akt phosphorylation. In combination, these results provided strong evidence that Exe also operates in sperm by activating the Akt axis. Furthermore, the decline in motility in parallel with viability following attenuation of Akt phosphorylation status by an inhibitor assigns a central role to the PI3K/Akt-dependent signaling pathway in the healthy status of these cells (“anti-apoptotic”). For the first time, we have postulated that insulin receptor substrate-1 (IRS-1) in human sperm could be phosphorylated at Ser312. This tyrosine-phosphorylated enzyme is fundamental for transducing insulin signals after receptor binding in somatic cells [9]. The presence of this substrate in sperm was not unforeseen; indeed, it might be a crucial molecular step in sperm development because insulin is expressed in and secreted from sperm, and it exerts an autocrine regulation of sperm metabolism and survival and it is a factor involved in the induction of capacitation [7,8]. In contrast, IRS-1 phosphorylated at Ser312 and c-Jun N-terminal protein kinase activation (p-JNK) are considered negative substrates in somatic cells because they induce insulin resistance and apoptotic alterations. Our results established, for the first time, that a time-dependent decline in sperm motility and viability was associated with increased levels of specific sites in the phosphorylation of these two enzymes. Moreover, here, we confirmed that TNF-α negatively affected sperm motility and viability in a concentration-dependent manner [13] and, previously, we demonstrated that sperm exposure to TNF-α led to an increase in chromatin damage and DNA fragmentation in a concentration- and time-dependent manner [13]. TNF-α and stressor stimuli exert their actions through the activation of multiple downstream signaling effectors, including JNK and IRS-1 Ser312 in somatic cells, resulting in alterations to cell proliferation, apoptosis, and insulin resistance [26,27,28,29,30]. TNF-α in sperm acts via the same mechanism; in fact, in this study, the addition of TNF-α resulted in stimulation of the p-46 and p-54 JNK isoforms at Thr1837/Tyr185 and of IRS-1 Ser312. To gain a global understanding of the interplay involving these three sperm signaling pathways, we examined the effects of Exe on the activity of these substrates. After sperm exposure to Exe, we showed, for the first time, that a pro-survival factor, such as GLP-1 mimetic, at least in part counteracts the proapoptotic action of TNF-α on sperm through partial downregulation of TNF-α-induced phosphorylation of p-IRS-1 Ser312 and p-JNK and reduced the time–increase levels of the two kinases. On the other hand, TNF-α exposure reduced basal p-Akt and Exe stimulated Akt phosphorylation, confirming similar findings in somatic cells [16,26,27]. So, GLP-1 protects human sperm from cellular damage via a mechanism involving the Akt, IRS-1, and JNK pathways, similar to other human and animal tissues. From our studies, it emerges that an imbalance involving the considered kinases, together with other factors, might occur in sperm from pathological subjects.

## 5. Conclusions

Here, we demonstrated in human sperm the involvement of a complex interplay among these three signaling substrates (“cross-talk”), as previously demonstrated for some somatic cell types [16,26]. More importantly, our data strongly suggested that increased phosphorylation of insulin receptor substrate-1 (p-IRS-1) at Ser312 and c-Jun N-terminal protein kinase (p-JNK) are an important causal element involved in diminishing sperm performance. Noteworthily, phosphorylated protein kinase B (p-Akt) is decreased and phosphorylated insulin receptor substrate 1 (p-IRS-1) Ser312 and phosphorylated c-Jun N-terminal protein kinase (p-JNK) are increased in skeletal muscle of patients with obesity, type 2 diabetes, and/or insulin resistance [31,32,33]. Consequently, if the same imbalance involving these kinases also arises in sperm, the induction of abnormal semen, as in asthenospermia commonly associated with these diseases [34,35,36] may eventuate. Biologically, this “cross-talk” between the pro-survival pathway and the activated negative signaling substrates in sperm might have potential beneficial therapeutic implications. Then, a better insight on the role of these downstream signaling pathways may allow the manipulation of such molecular mechanisms with potential and consequential therapeutic targeting implications worth exploring. Perhaps it might be useful to incubate semen with glucagon-like peptide (GLP-1) analogues when samples are used during IVF treatments and, therefore, it could be therapeutically exploited in the management of medically assisted procreation (ART) to improve sperm quality for better outcomes in ART cycles.

## Data Availability

The data sets analyzed in the present study are available from the corresponding author on reasonable and motivated request.
